# Estimands for factorial trials

**DOI:** 10.1002/sim.9510

**Published:** 2022-06-25

**Authors:** Brennan C. Kahan, Tim P. Morris, Beatriz Goulão, James Carpenter

**Affiliations:** ^1^ MRC Clinical Trials Unit at UCL London UK; ^2^ Health Services Research Unit University of Aberdeen Aberdeen UK

**Keywords:** 2 × 2, estimand, factorial trial, ICH‐E9 addendum, randomized controlled trial

## Abstract

Factorial trials offer an efficient method to evaluate multiple interventions in a single trial, however the use of additional treatments can obscure research objectives, leading to inappropriate analytical methods and interpretation of results. We define a set of estimands for factorial trials, and describe a framework for applying these estimands, with the aim of clarifying trial objectives and ensuring appropriate primary and sensitivity analyses are chosen. This framework is intended for use in factorial trials where the intent is to conduct “two‐trials‐in‐one” (ie, to separately evaluate the effects of treatments A and B), and is comprised of four steps: (i) specifying how additional treatment(s) (eg, treatment B) will be handled in the estimand, and how intercurrent events affecting the additional treatment(s) will be handled; (ii) designating the appropriate factorial estimator as the primary analysis strategy; (iii) evaluating the interaction to assess the plausibility of the assumptions underpinning the factorial estimator; and (iv) performing a sensitivity analysis using an appropriate multiarm estimator to evaluate to what extent departures from the underlying assumption of no interaction may affect results. We show that adjustment for other factors is necessary for noncollapsible effect measures (such as odds ratio), and through a trial re‐analysis we find that failure to consider the estimand could lead to inappropriate interpretation of results. We conclude that careful use of the estimands framework clarifies research objectives and reduces the risk of misinterpretation of trial results, and should become a standard part of both the protocol and reporting of factorial trials.

## 1 BACKGROUND

Factorial trials allow investigators to evaluate two or more interventions in a single study without need to increase the sample size.^1^, ^2^, ^3^, ^4^, ^5^ In a 2 × 2 factorial trial, patients are allocated to one of four groups: treatment A alone, B alone, both A and B, or neither A nor B. Then, all patients allocated to treatment A (A alone + A and B) can be compared against all patients who were not (B alone + neither A nor B), and similarly for treatment B. We call this a factorial (or at‐the‐margins) analysis.

However, factorial analyses rely on the assumption of no interaction (ie, that the effect of treatment A is the same whether the patient is allocated to treatment B or not), and can be biased when this assumption is violated.^1^, ^2^, ^5^, ^6^ Therefore, it is important to evaluate the plausibility of this assumption through interaction tests, and assess sensitivity to deviations from this assumption through appropriate sensitivity analyses.^2^, ^3^, ^4^, ^5^ A typical sensitivity analysis involves analyzing the trial as four separate groups (A alone, B alone, A and B, and neither A nor B), as this approach does not rely on the assumption of no interaction.^2^, ^3^, ^4^, ^5^ We call this a multiarm (or inside‐the‐table) analysis.^2^, ^5^, ^6^

However, the ideal main analysis and corresponding sensitivity analysis will depend on the specific aims of the trial. For instance, we may wish to know the effect of treatment A vs control either in the presence of treatment B or in its absence. While both these effects could be estimated using the same factorial estimator described above, they require different sensitivity analyses. Conversely, the effect of the combination of A and B against control would require both a different estimator and sensitivity analysis.

As such, understanding the key objectives of the trial is essential, both to allow the investigators to choose an appropriate main analysis and corresponding sensitivity analysis, and to allow readers to evaluate whether the chosen methods are appropriate to the research question. However, the key objectives of factorial trials are rarely reported,^5^ or else are stated using ambiguous terminology (eg, to understand the “independent” effects of treatments).

The recent ICH‐E9(R1) addendum on estimands^7^ provides a strategy to systematically think through the trial objectives and ensure study methods are aligned to the objectives. To achieve a precise definition of the treatment effect the trial aims to estimate, the estimand defines five components^7^, ^8^, ^9^, ^10^, ^11^, ^12^, ^13^, ^14^, ^15^: (i) population; (ii) treatment condition(s) of interest; (iii) outcome measure; (iv) population‐level summary (ie, how outcomes under different treatment conditions are to be compared); and (v) how intercurrent events, such as treatment discontinuation or switching, are to be handled.

In this article we: (i) define a set of estimands that could be used in factorial trials; (ii) describe main and sensitivity estimators; (iii) outline a framework for applying estimands to factorial trials; and (iv) demonstrate the framework through a re‐analysis of a published factorial trial. We focus on the setting where investigators want to do “two trials in one”, that is, where the aim is to evaluate the effects of both treatments A and B in the same trial, though we note the estimands defined here could equally be applied to the setting where only the effect of treatment A is of interest (eg, a two‐arm trial where treatment B is a concomitant treatment).

## 2 ESTIMANDS FOR FACTORIAL TRIALS

A complete estimand will require specification of the five components listed previously. Because the main differentiator of factorial trials is the use of additional treatments, we focus on the *treatment* component here, though we briefly discuss implications of the factorial design on the population‐level summary measure and the handling of intercurrent events. Other components (population, outcome) could be specified as they would in a conventional two‐arm design.

We consider the setting of a 2 × 2 factorial trial with treatments A and B. We focus on the comparison of treatment A to control.

When specifying an estimand for treatment A vs control, it may be tempting to define it solely in terms of treatment A, while ignoring treatment B. For instance, we may wish to specify the estimand as:

$$E\left(Y^{\left(Z_{A}=1\right)}-Y^{\left(Z_{A}=0\right)}\right)$$

where $Z_{A}$ denotes treatment A (1 = yes, 0 = no), and $Y^{\left(Z_{A}\right)}$ denotes the patient's potential outcome under treatment $Z_{A}$ (for instance, $Y^{\left(Z_{A}=1\right)}$ would denote the patient's potential outcome under $Z_{A}=1$). However, this definition is not valid, because the potential outcomes $Y^{\left(Z_{A}=1\right)}$ and $Y^{\left(Z_{A}=0\right)}$ are not well defined in this setting.^16^ This is because the value of $Y^{\left(Z_{A}\right)}$ may itself depend on whether the patient receives treatment B or not, that is, $Y^{\left(Z_{A}=0,Z_{B}=0\right)}$ may be different to $Y^{\left(Z_{A}=0,Z_{B}=1\right)}$ (where $Y^{\left(Z_{A}=0,Z_{B}=0\right)}$ denotes the patient's potential outcome under $Z_{A}=0$ and $Z_{B}=0$, where $Z_{B}$ refers to treatment B). Hence, an estimand written solely in terms of $Y^{\left(Z_{A}\right)}$ could in fact represent two different things.

Therefore, specifying the estimand only in terms of treatment A is not sufficiently clear, and it must be written in terms of treatment B as well. We can incorporate treatment B into the estimand in a number of different ways. For instance, we may wish to know the effect of treatment A vs control (i) in the absence of treatment B; (ii) in the presence of treatment B; (iii) when treatment B is given according to usual practice; or (iv) when used in combination with treatment B. Each of these questions represents a different treatment strategy, and hence corresponds to a different estimand (Table 1).

**TABLE 1:** Overview of estimands for factorial trials. $Z_{A}$ denotes treatment A (1 = yes, 0 = no), $Z_{B}$ denotes treatment B (1 = yes, 0 = no), ${z_{A}}^{\left(\mathrm{UP}\right)}$ and ${z_{B}}^{\left(\mathrm{UP},z_{A}=1\right)}$ denotes the value of $Z_{B}$ the patient would receive under usual practice with $z_{A}=1$ (and similarly for ${z_{B}}^{\left(\mathrm{UP},z_{A}=1\right)}$).

Estimand definition	Description
$\beta_{A,B=0}=E\left(Y^{\left(Z_{A}=1,Z_{B}=0\right)}-Y^{\left(Z_{A}=0,Z_{B}=0\right)}\right)$	Effect of treatment A in absence of treatment B (A alone vs control alone)
$\beta_{A,B=1}=E\left(Y^{\left(Z_{A}=1,Z_{B}=1\right)}-Y^{\left(Z_{A}=0,Z_{B}=1\right)}\right)$	Effect of treatment A in presence of treatment B (A + B vs control + B)
$\beta_{A,B=\mathrm{UP}}=E\left(Y^{\left(Z_{A}=1,Z_{B}\left(\mathrm{UP},Z_{A}=1\right)\right)}-Y^{\left(Z_{A}=0,Z_{B}\left(\mathrm{UP},Z_{A}=0\right)\right)}\right)$	Effect of treatment A when treatment B is given according to usual practice (A + usual practice B vs control + usual practice B)
$\beta_{A+B}=E\left(Y^{\left(Z_{A}=1,Z_{B}=1\right)}-Y^{\left(Z_{A}=0,Z_{B}=0\right)}\right)$	Effect of combination A and B (A + B vs control alone)

We formally define these estimands below. In subsequent sections we then specify how each of these estimands could be estimated. Note the estimands defined here are not intended to be comprehensive, and other estimands may also be of interest.

### 2.1 Estimand 1: Effect of treatment A in the absence of treatment B

This estimand is defined as the effect of treatment A vs control in the absence of treatment B (ie, if no one received treatment B):

(1) $$\beta_{A,B=0}=E\left(Y^{\left(Z_{A}=1,Z_{B}=0\right)}-Y^{\left(Z_{A}=0,Z_{B}=0\right)}\right).$$

### 2.2 Estimand 2: Effect of treatment A in the presence of treatment B

This estimand is defined as the effect of treatment A vs control in the presence of treatment B (ie, if everyone received treatment B):

(2) $$\beta_{A,B=1}=E\left(Y^{\left(Z_{A}=1,Z_{B}=1\right)}-Y^{\left(Z_{A}=0,Z_{B}=1\right)}\right).$$

### 2.3 Estimand 3: Effect of treatment A when treatment B is given according to usual practice

This estimand is defined as the effect of treatment A vs control if treatment B were given according to usual practice (ie, if patients received treatment B as they would under usual practice):

(3) $$\beta_{A,B=\mathrm{UP}}=E\left(Y^{\left(Z_{A}=1,Z_{B}\left(\mathrm{UP},Z_{A}=1\;\right)\;\right)}-Y^{\left(Z_{A}=0,Z_{B}\left(\mathrm{UP},Z_{A}=0\right)\;\right)}\right),$$

where $Z_{B}\left(\mathrm{UP},Z_{A}=1\right)$ is the potential value of $Z_{B}$ the patient would receive according to usual practice under $Z_{A}=1$, and vice versa for $Z_{B}\left(\mathrm{UP},Z_{A}=0\right)$.

This estimand may be of interest when the use of treatment B varies, so that some but not all patients would receive treatment B under usual practice.

### 2.4 Estimand 4: Effect of treatments A and B against control alone

This estimand is defined as the effect of treatment A when used in combination with treatment B (ie, the effect of A and B against neither treatment A nor B):

(4) $$\beta_{A+B}=E\left(Y^{\left(Z_{A}=1,Z_{B}=1\right)}-Y^{\left(Z_{A}=0,Z_{B}=0\right)}\right).$$

## 3 HANDLING OF INTERCURRENT EVENTS

Specification of how intercurrent events are to be handled is a key component of the estimand.^7^, ^10^, ^11^, ^12^, ^15^, ^17^, ^18^, ^19^ Intercurrent events are postrandomisation events which affect the interpretation or existence of the outcome measure, such as treatment discontinuation, treatment switching, or use of nontrial treatments such as rescue medication. The ICH‐E9(R1) addendum lists five strategies to handle intercurrent events: *treatment policy* (where the intercurrent event is taken to be part of the treatments being compared); *hypothetical* (where the treatment effect in a hypothetical scenario where the intercurrent event would not have occurred is of interest); *composite* (where the intercurrent event is incorporated into the definition of the outcome); *while‐on‐treatment* (where the outcome prior to the occurrence of the intercurrent event is of interest); and *principal stratum* (where the treatment effect in the principal stratum in which the intercurrent event would not occur is of interest).

Specification of how intercurrent events for treatment A (eg, discontinuation of treatment A) are to be handled is required for a complete estimand. However, because the factorial design means that treatment B is also part of the treatment strategy, additional specification of how intercurrent events related to treatment B are to be handled is required.

For instance, if we are interested in the estimand $\beta_{A,B=1}$ (the effect of treatment A in the presence of B), some patients may discontinue treatment B early, or may miss several doses. We would need to decide how this is handled in the estimand. For instance, a treatment policy strategy would address the question “the effect of treatment A vs control, when used alongside a policy of treatment B (regardless of whether people adhere to that policy).” Conversely, a hypothetical strategy would address the question “the effect of treatment A vs control, in a hypothetical scenario where everyone takes treatment B as intended.” The strategies for handling intercurrent events could in principle be different for A and B, for example, invoking a treatment policy strategy for treatment A with a hypothetical strategy for treatment B.

In some instances, treatment B itself may be a cause of intercurrent events for treatment A (for instance, if treatment B causes an adverse effect which necessitates discontinuation of all study treatments, including A). Here, if the estimand of interest is $\beta_{A,B=1}$ (the effect of treatment A in the presence of B), a treatment policy strategy may be most appropriate (as the intercurrent event would occur in practice, as patients are also taking treatment B); however, if the estimand $\beta_{A,B=0}$ (the effect of treatment A in the absence of B) is of interest, this intercurrent event would not occur in practice, as patients would *not* be taking treatment B. Hence, we may wish to use a hypothetical strategy for intercurrent events caused by treatment B (eg, the effect the hypothetical setting where patients did not discontinue A due to adverse events caused by treatment B), as this is more reflective of the effect seen in clinical practice where patients would not receive B. However, it can be difficult to ascertain causes of intercurrent events (ie, to determine whether treatment B was indeed the cause), so this decision should be handled with care.

## 4 POPULATION‐LEVEL SUMMARY MEASURE

The population‐level summary measure denotes how outcomes under different treatment conditions are to be compared (eg, through a difference in means, risk ratio, odds ratio, etc.). For collapsible summary measures^20^ (such as a mean difference, risk ratio, risk difference), the choice of summary measure could be made as in a parallel group trial. However, for noncollapsible summary measures (such as an odds ratio), additional considerations arise in a factorial trial (for further details on collapsible vs noncollapsible summary measures, see Reference 20).

The issue of noncollapsibility applies when patients can be grouped into different strata; then, a *conditional* estimand is based on the stratum‐specific effect, while a *marginal* estimand is based on the effect after collapsing over stratum (and, for noncollapsible summary measures, the values of these two estimands will typically differ). In a factorial trial, for the comparison of treatment A, patients may be grouped into stratum according to use of treatment B.

Consider the data in Table 2. Here, the odds ratio (OR) is 0.50 for treatment A, and 0.10 for treatment B, and there is no interaction on the log odds scale (ie, the OR for “A” vs “no A" is 0.50 both in the stratum of participants allocated to “B”, and the stratum allocated to “no B"). From this table, the conditional OR for treatment A vs control (conditional on whether patients were allocated to B), is 0.50; however, the marginal OR is 0.56. However, it is worth noting that true value of the marginal estimand for A depends on the distribution of treatment B. Hence, the value of 0.56 relates to a population where 50% of patients receive B; however, if 20% or 80% were to receive B, then this would lead to different marginal odds ratios.

**TABLE 2:** Event probabilities in a fictitious 2 × 2 factorial trial

		Treatment B	
		No	Yes
Treatment A	No	50%	9.1%
	Yes	33.3%	4.8%

Therefore, for the estimands defined earlier, we need to consider whether a marginal vs conditional interpretation is desired (if applicable; see below). We note the marginal vs conditional distinction also applies to baseline covariates (such as age, disease stage, etc.), however these same considerations apply to a two‐arm trial, so we do not consider this further here.

For estimands (1) and (2) (the effect of A in the absence of B, and the effect of A in the presence of B), we are interested in the treatment effect in the setting where no patients receive treatment B (or where all patients receive treatment B). Hence, all patients belong to the same stratum (because there is no variation in whether patients receive treatment B), and so the marginal vs conditional distinction does not apply, and so does not require specification in the estimand.

Similarly, for estimand (4) (the effect of the combination of A and B), the marginal/conditional distinction does not apply, as B is part of the treatment condition being compared.

For estimand (3) (the effect of treatment A when treatment B is given according to usual practice), the marginal/conditional distinction can apply (provided usual practice is not that either no patients, or all patients receive B), however this can be challenging in practice. In a factorial trial, patients belong to clearly defined strata (allocated to B, or not allocated to B), however in practice these strata may not be so well defined, for instance if $Z_{B}\left(\mathrm{UP},Z_{A}=1\right)\neq Z_{B}\left(\mathrm{UP},Z_{A}=0\right)$ (ie, if receipt of treatment B depends on use of treatment A). In this setting, a conditional estimand is not well defined. However, if $Z_{B}\left(\mathrm{UP},Z_{A}=1\right)=Z_{B}\left(\mathrm{UP},Z_{A}=0\right)$ (ie, if receipt of treatment B is independent of use of treatment A), then the strata are well defined, and so is the conditional estimand. In practice however, this will be difficult to ascertain, and so our view is that a marginal estimand is preferable, as the use of treatment B is generally not a typical baseline covariate, and so the conditional estimand may be ill‐defined.

## 5 CHOICE OF ESTIMAND

Different estimands may be of interest depending on the setting. If treatment B is a novel pharmaceutical which does not yet have regulatory approval, than interest may lie in the effect of treatment A in the absence of treatment B. Conversely, if treatment B is in common use, there may also be interest in the effect of treatment A in the presence of B, or when treatment B is given according to usual care.

In some situations, more than one estimand may be of interest, and multiple estimands could be defined. It may also be the case that which estimand is of most interest depends on the results of the trial itself. For instance, if the trial shows that treatment B is harmful, then the effect of treatment A in the absence of B will be most useful. Conversely, if treatment B is shown to be extremely effective, it may become the new standard of care, and so the effect of treatment A in the presence of B would be of interest. If more than one estimand is to be used, it is important to clarify which estimand(s) is/are considered primary.^21^, ^22^

Also of note, in certain settings the true value of different estimands will coincide. For example, when treatments A and B do not interact, then the true values of the estimands $\beta_{A,B=0}$ and $\beta_{A,B=1}$ are the same. Similarly, the estimand $\beta_{A,B=\mathrm{UP}}$ will coincide with either $\beta_{A,B=0}$ or $\beta_{A,B=1}$ if usual practice for treatment B is to withhold it from all patients (estimand $\beta_{A,B=0}$), or to give it to all patients (estimand $\beta_{A,B=1}$).

## 6 ESTIMATORS AND SENSITIVITY ANALYZES UNDER A FACTORIAL DESIGN

### 6.1 Factorial estimators

A factorial analysis is typically used for the primary analysis under a factorial design, as it is the most efficient approach, and enables the comparison of multiple interventions in a single study without need to increase the sample size. Here, the effect of treatment A is estimated by comparing all patients allocated to treatment A (treatment A alone + treatments A and B) against all those who did not (treatment B alone + neither treatment A nor B) and similarly for treatment B.

There are two ways in which this could be implemented. Under the first approach, the analysis model can be written as:

(5) $$Y=\alpha +\beta_{A}Z_{A}+\beta_{B}Z_{B}+\varepsilon ,$$

where Y is the observed outcome. Then, ${\hat{\beta }}_{A}$ and ${\hat{\beta }}_{B}$ are the factorial estimators for the effects of treatments A and B. Here, the effect of A is estimated while adjusting for the effect of B (and vice versa).

An alternative approach that is sometimes used is to omit the term $Z_{B}$ from the model to obtain an estimate for $\beta_{A}$ (and vice versa), for example, as:

(6) $$Y=\alpha +\beta_{A}Z_{A}+\varepsilon .$$

For collapsible effect measures (eg, difference in means, risk ratio, risk difference), the key difference between models (5) and (6) is efficiency; if treatment B affects outcome, then model (5) will be more efficient than (6) for the effect of A.^5^

However, for noncollapsible measures (eg, odds ratio), failure to adjust for the alternative treatment in the analysis (ie, using model (6)) can actually introduce bias for the estimands considered in this Article. This is because model (6) provides a marginal odds ratio based on the allocation ratio used in the trial (eg, a marginal OR if 50% of patients received treatment B, and 50% did not). However, this marginal OR does not match the any of estimands ((1), (2), (3), (4)) defined here, and hence will be biased. Conversely, model (5) (which adjusts for the alternative factor), will be unbiased for estimands (1), (2), and (4) under certain assumptions (see below), mainly that there is no interaction. This is, at first glance, counterintuitive, as model (5) appears to be estimating a conditional OR. However, if we consider estimand (1) as an example, we are interested in the effect of A in the absence of B. Model (5) can be seen to estimate the stratum‐specific OR (eg, in Table 2, this is 0.50); and, under the assumption of no interaction, this stratum‐specific OR of 0.50 is equal the OR in the group of patients who were not allocated to treatment B, that is, this estimator will be an unbiased estimator for the effect of A in the absence of B.

This will not be the case for estimand (3) (the effect of A when B is given according to usual practice), if interest lies in the marginal OR, and hence both models (5) and (6) will be biased for this. However, if interest lies in the conditional OR, model (5) will be unbiased under certain assumptions (Table 3).

**TABLE 3:** Assumptions required for unbiasedness for factorial^a^ designs

	Factorial analysis^b^		Multiarm analysis^c^	
Estimand	Estimator	Assumptions for unbiasedness	Estimator	Assumptions for unbiasedness
x	${\hat{\beta }}_{A}$	No interaction	${\hat{\beta }}_{A0}$	None
$\beta_{A,B=1}$	${\hat{\beta }}_{A}$	No interaction	${\hat{\beta }}_{\mathrm{AB}}-{\hat{\beta }}_{0B}$	None
$\beta_{A,B=\mathrm{UP}}$ ^d^	${\hat{\beta }}_{A}$	Assumes (1); no interaction; and (2) the decision to assign treatment B does not depend on whether they receive treatment A	$\left(1-\pi \right){\hat{\beta }}_{A0}+\pi \left({\hat{\beta }}_{\mathrm{AB}}-{\hat{\beta }}_{0B}\right)$ ^e^	Assumes (1) the decision to assign treatment B does not depend on whether they receive treatment A; and (2) estimators ${\hat{\beta }}_{A0}$ and ${\hat{\beta }}_{\mathrm{AB}}-{\hat{\beta }}_{0B}$ will be the same regardless of whether treatment B is assigned based on random allocation or according to usual practice
$\beta_{A+B}$	${\hat{\beta }}_{A}+{\hat{\beta }}_{B}$	No interaction	${\hat{\beta }}_{\mathrm{AB}}$	None

^a^ Based on a 2 × 2 factorial design with treatments A and B, where patients are randomly allocated (1) treatment A; (2) treatment B; (3) treatment A and treatment B; or (4) neither treatment A nor B (control).

^b^ Based on analysis model: $Y=\alpha +\beta_{A}Z_{A}+\beta_{B}Z_{B}+\varepsilon$

^c^ Based on analysis model: $Y=\alpha +\beta_{A0}Z_{A0}+\beta_{0B}Z_{0B}+\beta_{\mathrm{AB}}Z_{\mathrm{AB}}+\varepsilon .$

^d^ Assumptions for unbiasedness are based on a collapsible effect measure (eg, difference in means, risk ratio). For noncollapsible measures (eg, odds ratio), the estimators will be unbiased under the assumptions given for the conditional effect, however will not generally be unbiased for the marginal effect.

^e^ π is the proportion of patients assumed to receive treatment B in practice.

Table 3 shows the factorial estimators corresponding to each estimand, along with assumptions required for unbiasedness. We use ${\hat{\beta }}_{A}$ to denote the estimator from model (5) (and similarly for ${\hat{\beta }}_{B}$). The estimator ${\hat{\beta }}_{A}$ is used for the estimands $\beta_{A,B=0}$, $\beta_{A,B=1}$, and $\beta_{A,B=\mathrm{UP}}$, and the estimator ${\hat{\beta }}_{A}+{\hat{\beta }}_{B}$ is used for the estimand $\beta_{A+B}$.

The estimators above all require the assumption of no interaction for unbiasedness. The reason for this is that the factorial estimator, ${\hat{\beta }}_{A}$, targets the estimand $E\left(Y^{\left(Z_{A}=1,Z_{B}=z_{B,\text{Rand}}\right)}-Y^{\left(Z_{A}=0,Z_{B}=z_{B,\text{Rand}}\right)}\right)$, where $z_{B,\text{Rand}}$ denotes a random allocation to treatment B. This estimand is in itself usually not of direct interest, but its true value corresponds to those of estimands $\beta_{A,B=0}$, $\beta_{A,B=1}$, and $\beta_{A,B=\mathrm{UP}}$, when treatments A and B do not interact.

We note that the models above could also be adjusted for additional baseline covariates (such as age, disease stage, etc.), either to increase efficiency,^23^ or if a conditional estimand is desired.

### 6.2 Multiarm analyses (sensitivity analyses)

It is often difficult to rule out departures from the assumption of no interaction, as most trials are underpowered to assess this. Therefore, it is useful to perform sensitivity analyses which assess the impact of deviations to this assumption on results. Multiarm analyses can be unbiased even when treatments interact, and so make ideal sensitivity analyses. However, they have higher variance than the factorial estimators, owing to their smaller sample size, which is why they are typically not used for the primary analysis of factorial designs. We note here that we are using “sensitivity analysis” as defined in the ICH‐E9(R1) addendum (“*a series of analyses conducted with the intent to explore the robustness of inferences from the main estimator to deviations from its underlying modelling assumptions and limitations in the data"* ^7^), however alternative definitions are available.^24^

The model can be written as:

(7) $$Y=\alpha +\beta_{A0}Z_{A0}+\beta_{0B}Z_{0B}+\beta_{\mathrm{AB}}Z_{\mathrm{AB}}+\varepsilon ,$$

where $Z_{A0}$, $Z_{0B}$, and $Z_{\mathrm{AB}}$ denote treatment A alone, treatment B alone, or both treatments A and B.

Table 3 shows the multiarm estimators corresponding to each estimand, along with assumptions required for unbiasedness.

Because no patients are assigned to treatment B according to usual practice (unless usual practice is to give either no patients or all patients treatment B), the multiarm analysis cannot estimate $\beta_{A,B=\mathrm{UP}}$ without requiring additional untestable assumptions. However, it could still be used as a sensitivity analysis for the factorial analysis, as it makes *alternative* assumptions, and so if results between the two broadly agree, we can be more confident in our main conclusions.

The multiarm estimator for $\beta_{A,B=\mathrm{UP}}$ can be written as:

$${\hat{\beta }}_{A,\text{weighted}}=\left(1-\pi \right){\hat{\beta }}_{A0}+\pi \left({\hat{\beta }}_{\mathrm{AB}}-{\hat{\beta }}_{0B}\right),$$

where π is the proportion of patients assumed to receive treatment B in practice. This estimator separates the effect of treatment A according to the presence or absence of treatment B, then weights each component according to the assumed proportion of patients who would receive or not receive treatment B as part of usual practice.

It requires several assumptions for unbiasedness. First, it assumes that receipt of treatment B does not depend on use of treatment A (ie, that there are no patients who would receive treatment B as part of usual practice under treatment A but not under control, or vice versa). Second, because the patients who receive treatment B in a factorial trial are not the same as those who would receive treatment B as part of usual practice, this estimator relies on the assumption that the estimates ${\hat{\beta }}_{A0}$ and ${\hat{\beta }}_{\mathrm{AB}}-{\hat{\beta }}_{0B}$ will be the same regardless of whether treatment B is assigned based on random allocation or according to usual practice.

## 7 FRAMEWORK FOR IMPLEMENTING ESTIMANDS IN FACTORIAL TRIALS

A framework for implementing estimands in factorial trials is shown in Table 4. First, this involves specifying the estimand(s) of interest, including the handling of treatment B in the treatment component (whether interest is in the effect of treatment A in the absence of B, in its presence, etc.), as well as how intercurrent events related to treatment B will be handled.

**TABLE 4:** Framework for implementing estimands in factorial trials

1. Specify estimand(s) of interest, including specification of how the additional treatment(s) (eg, treatment B) will be handled in the treatment strategy, and how intercurrent events affecting the additional treatment(s) will be handled^a^.
2. Designate appropriate factorial estimator (adjusting for other factors) as primary analysis strategy
3. Report size of estimated interaction, alongside confidence interval and p‐value, to assess plausibility of assumption of no interaction underpinning factorial estimator
4. Perform sensitivity analysis using appropriate multiarm estimator to evaluate to what extent departures from the underlying assumption of no interaction may affect results

^a^ These should be specified alongside other components of the estimand, such as outcome, handling of intercurrent events related to treatment A, etc.

Second, a primary analysis strategy should be designated for each estimand. This would typically be based on the appropriate factorial estimator, as this provides the efficiency gains which typically motivate the use of the factorial design.

Third, the plausibility of the assumption of no interaction underpinning the factorial estimators should be assessed by evaluating the size of the estimated interaction term, along with confidence intervals and a *P*‐value. This can be done using the following model:

(8) $$Y=\alpha +\beta_{A}Z_{A}+\beta_{B}Z_{B}+\beta_{\mathrm{Int}}Z_{A}Z_{B}+\varepsilon ,$$

where ${\hat{\beta }}_{\mathrm{Int}}$ is the estimator for the interaction between treatments A and B. (note that this model should be used only to assess the interaction term, and not to test the terms $\beta_{A}$ or $\beta_{B}$ as part of a factorial analysis, as a model which includes the interaction term is equivalent to model (7) under different parameterization, and so will be equivalent to using a multiarm estimator, with the corresponding loss in precision).

Fourth, a sensitivity analysis using the appropriate multiarm estimator should be performed to evaluate to what extent results from the factorial estimators may be affected by departures from the underlying assumption of no interaction. Care should be taken when interpreting results of these sensitivity analyses, as some deviation between the multiarm estimators and factorial estimators is expected, due to random variation.

We also note that investigators sometimes perform a two‐stage analysis,^6^ where they perform an initial test of interaction; if the test is not significant at some predefined level, they perform a factorial analysis, and if it is significant, they perform a multiarm analysis. This approach has shown to be biased, and so we do not recommend it.^6^ In our view, a preferable approach is to use the test of interaction and multiarm analysis to assess the plausibility and sensitivity to the underlying assumption of no interaction, in line with recommendations in the ICH‐E9 addendum.^7^ By presenting both the factorial and multiarm estimators, alongside a formal assessment of the interaction, investigators and readers can judge for themselves whether they feel trial results are robust. However, we note this approach is not infallible; we will never know in truth whether an interaction exists or not (and hence, whether the main estimator is valid), and so the resulting conclusions will be, to some degree, subjective.

## 8 APPLICATION TO THE MIST2 TRIAL

We now use the previously published MIST2 trial as an illustrative example of how our framework could be applied. MIST2 was a 2 × 2 factorial trial which evaluated the use of two drugs (DNase and tPA) for patients with pleural infection.^25^ Here, we focus mainly on the evaluation of DNase, though the same considerations could equally apply to the evaluation of tPA. We focus on the outcome of referral for surgery at 3 months, which represents a failure of the intervention to improve symptoms.

### 8.1 Choice of estimand(s)

The first step during the trial design stage is to choose which estimand(s) is of interest. After deciding on the estimand, we can then choose the corresponding primary and sensitivity analyses. Neither DNase or tPA were in common use at the time the trial was designed (ie, both were “new” treatments for this condition), so the estimand $\beta_{A,B=0}$ (the effect of DNase in the absence of tPA; DNase alone vs placebo alone) would likely be of most clinical relevance, as this provides the effect of DNase as introduced into current clinical practice.

However, if tPA were found to be effective in this trial, it may then become part of standard clinical practice, in which case the estimand $\beta_{A,B=1}$ (the effect of DNase in the presence of tPA; DNase + tPA vs placebo + tPA) may also be of interest, as it provides the effect of DNAse if tPA were to be added to usual practice. This could therefore be included as a supplementary estimand (with $\beta_{A,B=0}$ being the primary estimand).

In this setting, since both DNase and tPA are new treatments, $\beta_{A,B=\mathrm{UP}}$ is the same as $\beta_{A,B=0}$ (ie, usual care is the absence of tPA), and so the estimand $\beta_{A,B=\mathrm{UP}}$ does not require additional consideration.

The estimand $\beta_{A+B}$ may also be of exploratory interest, and so could also be included as another supplementary estimand.

This leaves us with one main estimand ($\beta_{A,B=0}$) and two supplementary estimands ($\beta_{A,B=1}$ and $\beta_{A+B}$), and for each one, the other components comprising the estimand need to be defined. An example of how this could be done for the primary estimand is provided in Table 5.

**TABLE 5:** Example of how primary estimand for DNase vs placebo comparison could be written for the outcome referral for surgery in MIST2

Estimand component	Definition
Treatment conditions	DNase alone (without tPA) vs placebo alone (without tPA)
Population	Patients with pleural infection, as defined by the trial's inclusion/exclusion criteria
Outcome	Referral for thoracic surgery within 3 months of randomization
Population‐level summary measure	Marginal odds ratio
Intercurrent events	All intercurrent events related to DNase (failure to initiate treatment, treatment discontinuation, incorrect dose, etc.) will be handled using a treatment policy strategy
	Use of nontrial treatments (including tPA) will be handled using a treatment policy strategy
	Mortality will be handled using a while‐alive strategy (ie, the outcome is defined as whether the patient was referred for surgery within 3 months of randomization or before they died, whichever is sooner)

We note we are using an odds ratio as our population‐level summary measure, but that the conditional vs marginal distinction in relation to treatment B does not apply for the chosen estimands; however in Table 5 we have specified a marginal estimand in relation to any baseline covariates.

### 8.2 Analysis

The primary analysis for each of the three estimands could be based on the factorial estimator: ${\hat{\beta }}_{A}$ for estimands $\beta_{A,B=0}$ (effect of DNase alone) and $\beta_{A,B=1}$ (effect of DNase + tPA vs placebo + tPA), and ${\hat{\beta }}_{A}+{\hat{\beta }}_{B}$ for estimand $\beta_{A+B}$ (effect of DNase + tPA vs placebo) (where these estimators relate to the log(OR)). Importantly, this should be from model (5), that is, a model which also includes a treatment indicator for tPA as a covariate, given we are using an odds ratio.

Sensitivity analyses could be carried out using the corresponding multiarm estimators: ${\hat{\beta }}_{A0}$ for $\beta_{A,B=0}$, ${\hat{\beta }}_{\mathrm{AB}}-{\hat{\beta }}_{0B}$ for $\beta_{A,B=1}$, and ${\hat{\beta }}_{\mathrm{AB}}$ for $\beta_{A+B}$.

### 8.3 Results

For the primary estimand $\beta_{A,B=0}$ (effect of DNase alone), the OR under primary (factorial) estimator ${\hat{\beta }}_{A}$ was 2.44 (95% CI 1.06 to 5.65), denoting increased harm associated with DNase (Table 6). There was some evidence of an interaction (interaction OR 0.19; 95% CI 0.02 to 1.50; P‐value for interaction 0.12), though this was very imprecisely estimated (with a 75‐fold range between the lower and upper limit of the CI). Under a sensitivity analysis based on ${\hat{\beta }}_{A0}$, the OR was 3.46 (95% CI 1.32 to 9.02), which is consistent with the main results (albeit more extreme), highlighting that main results are likely robust to departures from the “no interaction” assumption.

**TABLE 6:** Estimates for the effect of DNase on referral for surgery in the MIST2 trial

Estimand	Estimator	Marginal odds ratio (95% CI)
DNase alone vs placebo alone ($\beta_{A,B=0}$)		
	Primary (factorial; ${\hat{\beta }}_{A}$)	2.44 (1.06, 5.65)
	Sensitivity (multiarm; ${\hat{\beta }}_{A0}$)	3.46 (1.32, 9.02)
DNase with tPA vs placebo with tPA ($\beta_{A,B=1}$)		
	Primary (factorial; ${\hat{\beta }}_{A}$)	2.44 (1.06, 5.65)
	Sensitivity (multiarm; ${\hat{\beta }}_{\mathrm{AB}}-{\hat{\beta }}_{0B}$)	0.65 (0.10, 4.09)
DNase with tPA vs placebo ($\beta_{A+B}$)		
	Primary (factorial; ${\hat{\beta }}_{A}+{\hat{\beta }}_{B}$)	0.34 (0.10, 1.21)
	Sensitivity (multiarm; ${\hat{\beta }}_{\mathrm{AB}}$)	0.23 (0.09, 0.40)

The supplementary estimand $\beta_{A,B=1}$ (effect of DNase + tPA vs placebo + tPA) could become quite relevant in this trial, as there was some evidence that tPA (treatment B in this example) was effective, which could lead to tPA becoming part of standard practice in the future (results for tPA: factorial estimate for the effect of tPA alone vs placebo OR 0.14; 95% CI 0.05 to 0.39; *P* < 0.001; sensitivity analysis 0.36 (0.09, 1.44). The primary (factorial) estimate for $\beta_{A,B=1}$ is ${\hat{\beta }}_{A}$ (as above); OR 2.44 (95% CI 1.06 to 5.65). However, the sensitivity analysis based on the multiarm estimator ${\hat{\beta }}_{\mathrm{AB}}-{\hat{\beta }}_{0B}$ provides contradictory results (OR 0.65; 95% CI 0.10 to 4.09), highlighting that inferences for this estimand are highly sensitive to the assumption of no interaction.

Results for the estimand $\beta_{A+B}$ (effect of DNase + tPA vs placebo) under a primary (factorial) estimator (${\hat{\beta }}_{A}+{\hat{\beta }}_{B}$) denote some evidence of benefit (OR 0.34; 95% CI 0.10 to 1.21), and the sensitivity analysis based on the multiarm estimator ${\hat{\beta }}_{\mathrm{AB}}$ showed consistent results (OR 0.23; 95% CI 0.09 to 0.40).

## 9 DISCUSSION

Factorial trials offer an efficient method of evaluating multiple interventions within a single trial. However, the use of additional interventions can obscure the exact treatment effect investigators wish to evaluate. To obviate this, we argue that careful use of the estimands framework clarifies the research objectives, highlighting exactly which treatment effects are to be estimated, and can guide investigators in choosing appropriate main and sensitivity analyses.

The key difference in specifying the estimand in a factorial trial compared to a standard two‐arm design is explanation of how additional treatments are to be handled in the treatment components of the estimand (eg, are we interested in the effect of treatment A in the absence of B? In its presence? When it is given according to usual practice?), and how intercurrent events related to additional treatments (eg, discontinuation) are to be handled.

We propose a simple framework to apply estimands to factorial trials (Table 4), which involves specifying the estimand(s) of interest (including how the additional treatment(s) are to be handled); identifying an appropriate primary analysis strategy (typically based on a factorial analysis, to realize the efficiency gains of the factorial design); assessing the interaction, to evaluate the plausibility of the assumptions underpinning the primary estimator; and conducting the appropriate multiarm sensitivity analysis to evaluate the robustness of the primary analysis to departures from its underlying assumptions.

Consideration of the target estimand at the design stage can also help determine whether a factorial design is the most appropriate choice. For instance, if interest is in several treatment effects (eg, the effect of treatment A both in the presence and absence of treatment B), a factorial design allows estimation of both. However, if interest were mainly in the effect of treatment A if treatment B were given according to usual practice, then a two‐arm parallel group design testing A vs control, which allows treatment B to be given according to usual practice, may be a preferable choice as it requires fewer assumptions than a factorial design.

Our main focus in this article is on “2‐for‐1” factorial trials, however we note the estimands used here could also be useful in trials where interactions are both expected and of primary interest, or indeed in simple two‐arm trials, where patients may receive multiple background therapies in addition to the treatments being evaluated. The use of such therapies is often included in the protocol (as stipulated by the SPIRIT guidelines^26^), but it can be useful to include them explicitly in the estimand in order to clarify the exact treatment conditions being compared (which can be particularly useful if the use of such background therapies are expected to modify the treatment effect, and their use in usual practice varies across settings).

In this article we have used the principles outlined in ICH‐E9^7^ to develop our proposed framework for implementing estimands into factorial trials. Our framework was developed with trials of healthcare interventions in mind, where unbiased estimation of benefits and harms is imperative to aid in medical decision making. However, in other settings, efficiency may be as important, in which case investigators may be comfortable using the factorial estimator even in presence of mild interactions due to efficiency gains (ie, lower mean‐squared error). The appropriateness of each of these approaches will depend on the specific trial aims.

In this article we have recommended using model (5) to implement a factorial analysis, though an alternate perspective is to use model (8) which incorporates the interaction term. As noted, this model is equivalent to model (7), and so the individual parameters from this model cannot be used for a factorial analysis, however investigators could use a combination of parameters to obtain a factorial‐style estimator.

## 10 CONCLUSION

Careful use of the estimands framework clarifies research objectives and reduces the risk of misinterpretation of trial results (which is otherwise increased in a factorial trial because of the complications of the two or more concurrent treatments). We therefore argue that using estimands in this way should become a standard part of both the protocol and reporting of factorial trials.

## AUTHOR CONTRIBUTIONS

Brennan C. Kahan wrote the first draft of the manuscript and analyzed the data. Tim P. Morris, Beatriz Goulão, and James Carpenter revised the manuscript. All authors read and approved the final manuscript.

## FUNDING INFORMATION

Brennan C. Kahan, Tim P. Morris, and James Carpenter are funded by the UK MRC, Grants No. MC_UU_00004/07 and No. MC_UU_00004/09.

## CONFLICT OF INTEREST

The authors declare that there is no conflict of interest.

## Data Availability

The data used for re‐analysis of the MIST2 trial were obtained from the tables in the original publication in Reference 25).
